# The Implication of Horizontal Gene Transfer Between *Acanthamoeba* and Its Intracellular Microbes on Pathogenicity: A Systematic Review

**DOI:** 10.3390/pathogens15060610

**Published:** 2026-06-08

**Authors:** Yalewayker Asrat, Biruk Bayleyegn, Mark Willcox, Nicole Carnt, Binod Rayamajhee

**Affiliations:** 1 School of Optometry and Vision Science, Faculty of Medicine and Health, University of New South Wales, Sydney, NSW 2052, Australiam.willcox@unsw.edu.au (M.W.); binod.rayamajhee@mq.edu.au (B.R.); 2 Faculty of Medicine, Health and Human Sciences, Macquarie University, Sydney, NSW 2109, Australia; 3 Department of Infection and Immunology, Kathmandu Research Institute for Biological Sciences (KRIBS), Kathmandu 44700, Nepal

**Keywords:** amoeba-resisting microorganisms, lateral gene exchange, endosymbionts, virulence factors, antimicrobial resistance

## Abstract

Background: *Acanthamoeba* is a free-living protozoan widely distributed in the environment and causes *Acanthamoeba* keratitis, skin, and brain disease. *Acanthamoeba* can exchange genes, potentially increasing antimicrobial resistance and virulence. Therefore, this systematic review aimed to summarize published studies on horizontal gene transfer (HGT) between *Acanthamoeba* and its intracellular microorganisms and to evaluate the impact of HGTs on the pathogenicity of *Acanthamoeba*. Methods: This systematic review was conducted following the recommended reporting guidelines of the Preferred Reporting Items for Systematic Reviews and Meta-analysis (PRISMA) statement guideline. The electronic databases PubMed, Embase, and Web of Science were used to search for relevant published research articles. Results: Nineteen studies that fulfilled the inclusion criteria were included in this systematic review. A total of 14 (73.6%) studies reported evidence of HGT involving *Acanthamoeba*, and five studies of the nineteen (26.3%) analysed the presence of intracellular microorganisms on the pathological effects of the host *Acanthamoeba*. Horizontally transferred genes were predominantly reported from *Pseudomonas* species, *Legionella pneumophila*, and *Chlamydia* species. Conclusions: HGT can occur among intracellular microorganisms and their host *Acanthamoeba*. *Acanthamoeba* harbouring intracellular microbes showed enhanced pathogenic effects on human corneal epithelial cells and in a mouse model. However, heterogeneity among the included studies precluded meta-analysis. Studies using clinical and environmental samples are needed to characterize the horizontal transfer of virulence and antimicrobial resistance genes.

## 1. Introduction

*Acanthamoeba* is an opportunistic free-living protozoan that is widely distributed across diverse natural environments, including seawater, swimming pools, tap water, natural thermal waters, soil, dust, and the nasal mucosa of healthy individuals [1]. *Acanthamoeba* is known to cause a painful sight-threatening corneal infection known as *Acanthamoeba* keratitis (AK), as well as granulomatous amoebic encephalitis (GAE) and skin infections [2]. Whilst most of these tend to occur in immunocompromised individuals [2], AK also occurs in healthy contact lens wearers.

**Figure 1:**
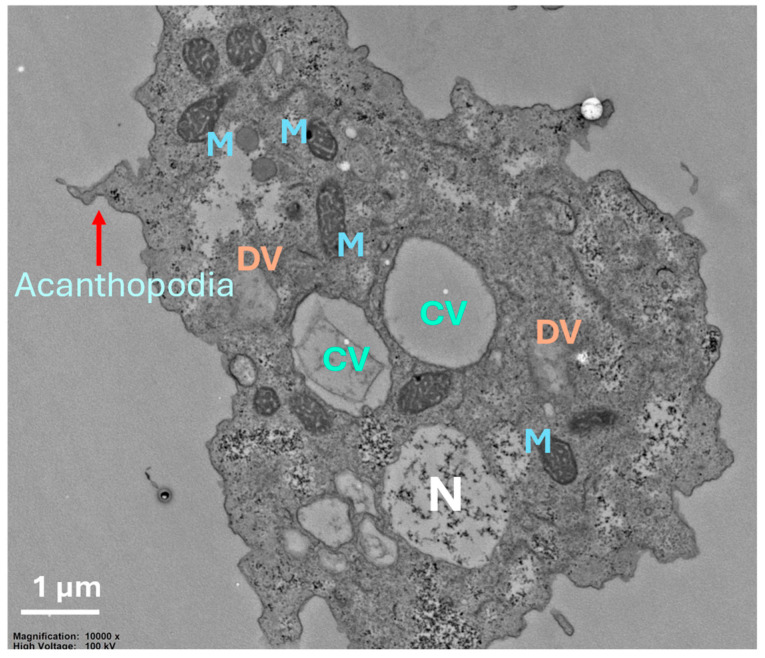
Transmission electron microscopy (TEM) image of an *Acanthamoeba* trophozoite. The trophozoite is characterized by multiple finger-like acanthopodia projecting from its surface (indicated by the arrow). The large, clear vesicle in the cytoplasm is the contractile vacuole (CV). Other cellular structures include the digestive vacuole (DV), nucleus (N), and mitochondria (M). This image was kindly provided by Binod Rayamajhee, UNSW Sydney, licensed under CC BY 4.0 [40]. Scale bar represents 1 μm.

**Figure 2:**
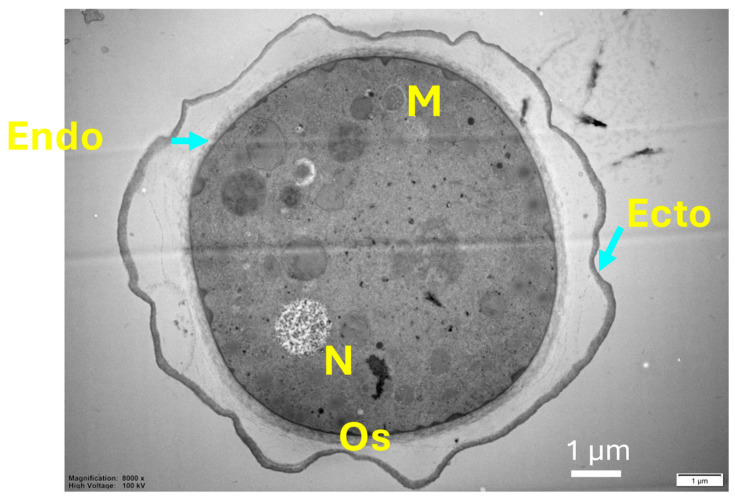
Ultrastructure of an *Acanthamoeba* cyst. The cyst is characterized by a double wall, consisting of an outer wrinkled wall (ectocyst) and an inner wall (endocyst). The endocyst and ectocyst join closely at the ostiole region (Os). The inter-cystic space is the region located between the outer ectocyst and inner endocyst layers of the cyst wall. Indicators: Os, ostiole; M, mitochondria; and N, nucleus. This TEM image adapted from Binod Rayamajhee’s PhD thesis, UNSW Sydney, licensed under CC BY 4.0 [40]. Scale bar represents 1 μm.

Depending on environmental conditions, *Acanthamoeba* exists in two transitional life-cycle stages: the trophozoite stage (Figure 1) and the cyst stage (Figure 2) [3]. The trophozoite stage is metabolically active and infective, and during this stage, the amoebae feed on bacteria and other microbes, multiply, and can invade host cells [4]. Cysts are the dormant environmentally resistant stage of *Acanthamoeba*, enabling long-term survival under adverse conditions [5]. *Acanthamoeba* species have traditionally been classified into three morphological groups [6]. Group I is characterized by large cysts (>18 μm), Group II comprises smaller cysts (<18 μm) and Group III includes cysts generally smaller than 19 μm, with globular endocysts and smooth or slightly wavy ectocysts [6,7]. According to this traditional classification, most pathogenic *Acanthamoeba* belong to group II [8]. A more advanced classification of *Acanthamoeba* is genotype assignment using small subunit ribosomal RNA (18S rRNA) sequences [9]. Based on this classification, *Acanthamoeba* comprises 23 genotypes (T1–T23), with genotype T4 being the most frequently identified in both clinical and environmental isolates [10].

Horizontal gene transfer (HGT) is the non-sexual transfer of genetic information between genomes through mechanisms other than parent–offspring inheritance [11]. It is a major driver of genome evolution, phenotypic diversity, and the expansion of protein families, thereby contributing to the emergence of new metabolic pathways and cellular traits [12]. HGT differs from vertical gene transfer, which is the standard transmission of genetic material from parent to offspring. Whole-genome sequencing studies have revealed that HGT is a major evolutionary force driving prokaryotic evolution [13]. HGT plays a significant role in the acquisition of key biological traits. For example, bacteria can acquire drug resistance from other bacteria during their lifetime via HGT [14].

*Acanthamoeba* are phagocytic protists that feed on microbes, including bacteria, fungi, and algae, by selective grazing, thereby helping to regulate microbial populations in the environment [15,16]. Moreover, *Acanthamoeba* species can harbour human pathogenic microorganisms such as *Legionella* and *Mycobacteria* spp., as well as giant viruses, which it may not consume but rather provide protection against environmental threats such as antibiotics, immune responses, and other external pressures [17]. Recent research has increasingly focused on understanding the interaction of *Acanthamoeba* with their intracellular microbiota, particularly due to their complex predatory behaviour and significance in microbial ecology. This area of research has expanded to explore the genetic and molecular mechanisms underlying these interactions and to show their evolutionary and ecological importance [18].

Studies have reported gene transfer among different bacteria species residing within *Acanthamoeba*, suggesting that *Acanthamoeba* may play a role in the development of drug resistance and the development of virulence traits by facilitating the exchange of genes between bacterial species [19]. Pathogenic bacteria that can both survive and replicate within *Acanthamoeba* include *Legionella pneumophila*, *Escherichia coli*, *Pseudomonas aeruginosa*, *Vibrio cholerae*, *Listeria monocytogenes*, *Mycobacterium avium*, and *Salmonella typhimurium* [20]. The ability for bacteria to survive inside *Acanthamoeba* may train the bacterial endosymbionts to also withstand phagocytosis by white blood cells during infection [21].

Amoeba-resisting viruses (ARVs), such as Pandoraviruses and *Acanthamoeba castellanii* medusavirus, have been isolated from hot spring water and were shown to survive in *Acanthamoeba castellanii* [22]. Giant viruses, *Adenovirus*, and *Coxsackievirus* have also been reported to survive in the intracellular cytoplasm of *Acanthamoeba* host cells [23,24,25,26]. *Acanthamoeba* has been suggested to be the natural host of *Mimivirus*, as genomic analyses indicate that most eukaryote-derived horizontally transferred genes in *Mimivirus* were likely acquired from amoebae [27]. Additionally, the intracellular survival and replication of a novel virus, *Yaravirus brasiliensis*, within *A. castellanii* have been reported, suggesting that amoebae may serve as a suitable host that supports viral survival and multiplication [25].

*A. castellanii* can serve as an important environmental reservoir and potential host for several endemic fungal pathogens, including *Histoplasma capsulatum*, *Cryptococcus* spp., *Blastomyces dermatitidis*, and *Sporothrix schenckii* [28]. It has the ability to interact with, harbour, and potentially protect these fungi in environmental settings, and contribute to their intracellular survival and transmission [28,29]. The presence of *Gloeotinia* spp. as a new fungal endosymbiont in clinical *Acanthamoeba* isolates has been reported [30]. This interaction may influence the pathogenicity of *Acanthamoeba* and could contribute to the increased virulence of the associated microorganisms [31]. Similarly, in a microbiome study of *Acanthamoeba* isolates recovered from patients with AK, intracellular survival of the fungus *Malassezia restricta* has been reported [32].

This systematic review aims to summarize published studies on HGT between *Acanthamoeba* and its intracellular microorganisms and to evaluate the impact of this phenomenon on the pathogenicity of *Acanthamoeba* and the intracellular microorganisms during human infections. Its findings will be useful in understanding the pathogenic factors of *Acanthamoeba* and the acquisition of new virulence factors by bacteria. Understanding the HGT of antimicrobial resistance genes between bacteria within *Acanthamoeba* highlights an under-studied area of HGT and will assist in the understanding of how these genes are acquired.

### 1.1. General Characteristics of Free-Living Amoeba Affecting Human Health

Free-living amoebae (FLA) are ubiquitous protozoa found in a wide variety of environmental habitats, such as soil, freshwater, seawater, and dust [33]. Pathogenic FLA genera, including *Acanthamoeba* spp., *Naegleria fowleri*, *Balamuthia mandrillaris*, *Sappinia diploidea*, *Vermamoeba vermiformis*, and *Vahlkampfia* spp., are recognized as causative agents of human disease, differing in their tissue tropism, pathogenesis, and clinical manifestations (Table 1). *Acanthamoeba* is exceptional among the pathogenic free-living amoebae in causing both amoebic keratitis (AK) and fatal granulomatous amoebic encephalitis (GAE), whereas *Naegleria fowleri* causes primary amoebic meningoencephalitis (PAM), which is a rapid fatal infection of the central nervous system, and *Balamuthia mandrillaris* is primarily associated with GAE [33,34,35].

**Table 1:** Pathogenic free-living amoebae (FLA) affecting humans and their general characteristics.

Reference	Species of FLA	Major Clinical Manifestations	Organ(s) Affected	Pathogenicity	Differential Diagnosis
Wang et al., 2023 [1]	*Acanthamoeba* spp.	*Acanthamoeba* keratitis (AK), granulomatous amoebic encephalitis (GAE), skin and lung infection	Cornea, conjunctiva, skin, lung, CNS (central nervous system)	The pathogenicity is mediated by adhesins, including mannose-binding protein (MBP) and laminin-binding protein (LBP), and the production of proteases, phospholipases, and cytolytic molecules that can trigger phagocytosis and cause pathological damage to mammalian cells.	Bacterial keratitis, fungal keratitis, herpes simplex keratitis, *Balamuthia*-associated GAE
Schuster et al., 2004 [36]	*Naegleria fowleri*	Primary amoebic meningoencephalitis (PAM)	CNS	Highly virulent, can cause death within 1–2 weeks of hospitalization; enters through the nasal cavity by penetrating the mucosal epithelial layer and migrates to the brain via olfactory nerves. Pathogenic determinants include the secretion of enzymes such as phospholipase and neuraminidase, and the formation of pores in target cell membranes, which can promote cell lysis and enhance phagocytic activity.	Acute bacterial meningitis, viral encephalitis
Visvesvara et al., 2007 [33]	*Balamuthia mandrillaris*	GAE, cutaneous lesions	CNS, skin	GAE can have high mortality. Stimulates brain microvascular endothelial cells to secrete the pleiotropic cytokine interleukin-6, a mediator of the early inflammatory response. Metalloprotease activity may facilitate extracellular matrix degradation. Interacts with extracellular matrix components, including collagen I, fibronectin, and laminin-1.	*Acanthamoeba* GAE, tuberculosis, fungal CNS infection, brain tumours
Siddiqui et al., 2024 [37]	*Sappinia diploidea*	A non-granulomatous amoebic encephalitis	CNS	A rare human pathogen. The pathogenesis of *Sappinia* species remains unclear because only a single human case of *Sappinia*-associated amoebic encephalitis has been documented. Experimental studies have shown that *Sappinia* is capable of infecting both immunodeficient and immunocompetent mice.	Brain abscesses, bacterial and fungal encephalitis
Siddiqui et al., 2021 [38]	*Vermamoeba vermiformis*	Keratitis, rare opportunistic parasitic infection in a patient with meningoencephalitis and bronchopneumonia	Cornea, respiratory tract	Limited information on the pathogenesis of *V. vermiformis*. Can cause host cell damage through trogocytosis (piecemeal phagocytosis) and the secretion of cytopathic factors.	*Acanthamoeba* keratitis, bacterial keratitis
Kinnear et al. [39]	*Vahlkampfia* spp.	Rare keratitis and opportunistic infections	Cornea	Pathogenesis is poorly understood. May involve adherence to corneal epithelial cells and direct cytopathic damage to host tissues.	*Acanthamoeba* keratitis, fungal keratitis

Key: central nervous system = CNS, granulomatous amoebic encephalitis = GAE, laminin-binding protein = LBP, mannose-binding protein = MBP, primary amoebic meningoencephalitis = PAM.

### 1.2. Mechanisms of Gene Transfer

Transduction, which is one of the mechanisms of gene transfer, involves virus-mediated transfer of genetic material [41]. Transformation involves the uptake of free DNA from the environment, whereas conjugation involves the direct exchange of plasmids between prokaryotes through a pilus [42]. Gene transfer can also occur from prokaryotes to eukaryotes, often facilitated by cell ingestion [43]. HGT in eukaryotes involves multiple key steps: the introduction of foreign DNA or RNA into the recipient cell, its transport into the nucleus, reverse transcription in the case of RNA, and subsequent integration into one or more host chromosomes. This integration can occur accidentally via the host’s DNA repair and recombination pathways or through an active integration mechanism [44]. Viral genes found in eukaryotic genomes have been associated with distinct cellular processes, with most of the HGT events between viruses and eukaryotes predicted to function in the cytoplasm, followed by the nucleus, mitochondria, and extracellular space [45].

**Figure 3:**
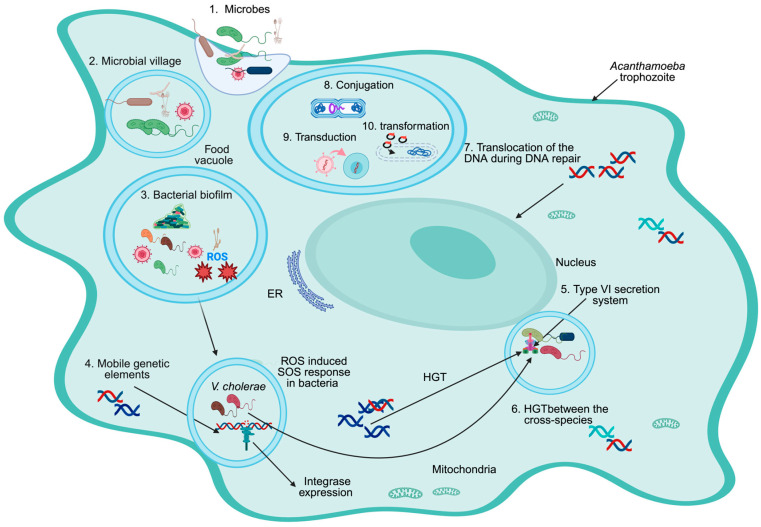
An illustrative diagram depicting multidimensional horizontal gene transfer (HGT) of intracellular microorganisms within the *Acanthamoeba* host (https://BioRender.com/eruv02d, accessed on 7 May 2026). 1. The phagotrophic feeding mechanism of *Acanthamoeba* promotes continual exposure to the foreign DNA of bacteria, viruses, and fungi, and supports intracellular persistence [21]. 2. This nonselective predation behaviour of *Acanthamoeba* results in sympatric bacteria within the same food vacuole, as in a ‘microbial village’. 3. Bacterial biofilm and oxidative stress from reactive oxygen species (ROS) can induce the SOS response in bacteria, such as *Vibrio cholerae*. 4. *Vibrio cholerae* enhances integron-integrase expression and promotes integration of mobile genetic elements/gene cassettes [47]. 5. It has been reported that *V. cholerae* employs a type VI secretion system (T6SS) to uptake other bacterial DNA (free mobile dsDNA or short ssDNA) in the food vacuole [48]. The flux of genetic elements in multiple directions assists transformation in bacteria via integration with SOS-regulated DNA/mobile genetic elements (MGEs) [49]. 6. This highlights the role of *Acanthamoeba* as an evolutionary hub for the emergence of new microbes, facilitating horizontal gene exchange between the cross-species [50]. This happens during their intracellular survival of bacteria, viruses, and fungi in the *Acanthamoeba* host [51]. 7. Translocation of the DNA and horizontal gene transfer between amoeba-resisting intracellular microorganisms and the host *Acanthamoeba,* either during the host DNA repair and recombination pathway or an active integration mechanism [44,52,53]. 8. The presence of plasmids in *Acanthamoeba*, an obligate intracellular microbial, suggests that conjugation contributes to horizontal gene transfer among rickettsial endosymbionts [50]. 9. Virus-mediated horizontal gene transfer occurs via transduction among intracellular microbes within the *Acanthamoeba* host [43]. 10. Transformation of genes occurs as a result of the SOS response and integron-integrase expression among intracellular microbes within *Acanthamoeba* [54]. The intimate association of microorganisms within *Acanthamoeba* provides a conducive environment for horizontal gene transfer among co-existing microbes [55,56].

Owing to *Acanthamoeba’s feeding* behaviour, various microorganisms can end up coexisting within the same food vacuole, potentially enabling interactions and genetic exchanges between intracellular microbes [21]. Moreover, studies have shown that phagotrophic organisms exhibit a significantly higher rate of HGT compared to non-phagotrophic organisms [46] (Figure 3).

## 2. Materials and Methods

### 2.1. Protocol Registration and Design

The principal aim of this systematic review was to describe the type of genes transferred between *Acanthamoeba* and its intracellular microorganisms, and within its endosymbionts. A secondary objective was to assess how HGT affects the pathogenesis of *Acanthamoeba* and its endosymbionts. This systematic review was registered in PROSPERO (International Prospective Register of Systematic Reviews) (CRD420251103780). An extensive search for existing systematic reviews on this topic was conducted to avoid duplication.

### 2.2. Databases and Search Strategy

The recommended reporting guideline of the Preferred Reporting Items for Systematic Reviews and Meta-analyses (PRISMA) statement guideline was followed to report this systematic review (Supplementary File S1) [57]. A systematic search of the electronic databases PubMed, Embase and Web of Science was carried out for relevant published research articles on HGT between *Acanthamoeba* and its intracellular microorganisms. Research articles published in the English language between September 1998 and February 2025 were included. Using Medical Subjects Headings (MeSHs), relevant articles for this systematic review were retrieved. The search terms or phrases used for retrieving published research articles included: “Gene transfer, Horizontal”, “Lateral gene transfer”, “*Acanthamoeba*”, “*Acanthamoeba castellani*”, “Free living amoebae”, “Intracellular *Acanthamoeba* endosymbionts”, “Bacterial endosymbionts”, “Virulence” and “Amoeba resisting bacteria”. The search strings were implemented using the Boolean operators “OR” or “AND”.

### 2.3. Inclusion and Exclusion Criteria

In this systematic review, all searched articles on HGT between *Acanthamoeba* species and its intracellular endosymbionts were included if studies had the following inclusion criteria: original research articles published in English from September 1998 through February 2025 and available in full text. Short communication reports, conference abstracts, reviews, research articles not published in English, studies that did not assess HGT involving *Acanthamoeba* species, and studies with insufficient methodological detail to determine whether HGT had been evaluated were not included.

### 2.4. Search Methods, Data Extraction and Quality Assessment

Duplicate records were removed using EndNote reference manager version 21 (Clarivate, Philadelphia, PA, USA). Then, two reviewers independently screened the abstracts and titles to determine which articles proceeded to full text review. Full-text articles were assessed for eligibility based on the above predefined inclusion and exclusion criteria. Disagreements between reviewers in the inclusion and quality of each research article were resolved through consensus discussion with the entire author team. In addition, a snowball referencing method was used by reviewing the reference lists of the included research articles to identify any additional studies, which were then evaluated for inclusion by title and abstract review. The quality of all of the included studies in this systematic review was assessed by the two authors using the Joanna Briggs Institute (JBI; University of Adelaide, SA, Australia) quality appraisal checklist (Table S1) [58].

## 3. Results

### 3.1. Results of the Articles Search

**Figure 4:**
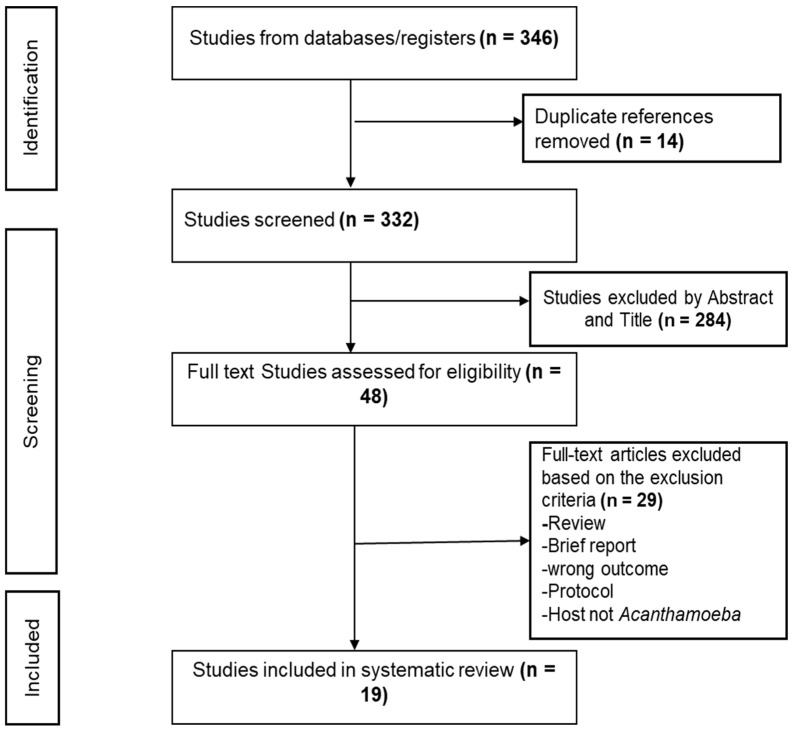
Preferred Reporting Items for Systematic Reviews and Meta-analyses (PRISMA) flow diagram for selection of articles included in the systematic review.

Initially, 346 published articles were identified through electronic database searches. A total of 14 duplicates were removed. After that, the remaining 332 studies were screened by their titles and abstracts, and during this process, 284 articles were removed. Finally, after full-text evaluation of 48 articles, 19 were found to be eligible for inclusion in this systematic review, and data were extracted using the standardized data extraction sheet (Table S2). The overall screening and eligibility steps and the number of articles selected at each step are described in the figure below (Figure 4).

### 3.2. Characteristics of Included Studies

The 19 included studies were published from 1998 [59] to 2025 [60]. The host *Acanthamoeba* species, identity of *Acanthamoeba* endosymbionts, the number of samples with intracellular microbes, the types of genes transferred, and the HGT method utilized were examined.

### 3.3. Evidence of HGT in the Acanthamoeba Host

A total of 14 studies reported evidence of HGT involving *Acanthamoeba*. Laboratory and computational methods used for HGT detection included whole-genome comparative analyses, phylogenetic reconstructions, genomic annotation with BLAST analysis using Rapid Annotation using Subsystem Technology (RAST), and functional annotation using the Kyoto Encyclopedia of Genes and Genomes (KEGG) [52,53,56]. Most studies reported HGT predominantly in genotype T4 of the *Acanthamoeba* species host from both the environmental and clinical isolates [52,53,55,61,62,63,64,65].

HGT occurred predominantly from amoeba-resisting intracellular microorganisms, including *Pseudomonas* species, *Legionella pneumophila*, *Legionella drancourtii*, *Chlamydiae* species, *Mycobacterium* species, *Vibrio cholerae*, *Klebsiella* species, *Burkholderia* species, *Aspergillus* species, and giant DNA viruses (*Pandoraviridae*, *Mimiviridae*, *Medusavirus*, and *Marseillevirus*), to the *Acanthamoeba* host [52,53]. Five of the included studies found HGT between *Acanthamoeba* species and *Pseudomonas* species [52,60,63,66]. Five studies also reported gene transfer from *Legionella* species to the *Acanthamoeba* host [52,63,67,68]. Two studies demonstrated genes had likely been transferred from *Mycobacterium* species [52,63]. The possibility of gene transfer from the genus *Chlamydia* to the *Acanthamoeba* host was reported by four studies [52,55,63,65]. One study demonstrated gene transfer between the phyla of Chlorobacteria, Cyanobacteria, and Firmicutes and the *Acanthamoeba* host [65].

Four studies reported HGT from the giant viruses in the families or genera of *Pandoraviridae*, *Medusavirus*, *Mimiviridae*, *Marseilleviridae*, *Pithoviridae*, and *Molliviridae*, to the host *Acanthamoeba* [52,53,61,62]. However, a study investigating lateral gene transfer between *Lausanne virus* and *A. castellanii* strain ATCC 30010 found no evidence of gene transfer [61] (Table 2).

### 3.4. Genes Horizontally Transferred Between the Acanthamoeba Host and Its Intracellular Microorganisms and Vice Versa

One study analysed seven clinically isolated *Acanthamoeba* species and demonstrated bidirectional gene transfer, including virulence-associated genes (VAGs) such as metalloproteases, cysteine proteases, laminin-binding proteins (LBP), heat-shock proteins (HSP), as well as metabolic and signalling genes and viral homologs, from amoeba-resisting microorganisms which can live in *Acanthamoeba*, including giant viruses, to *Acanthamoeba* species of genotypes T4 and T3 [52]. In a whole-genome comparative genomic study of *Acanthamoeba triangularis* strain SH 621, 99 *Acanthamoeba triangularis* genes showed the best BLASTp hits to amoeba-resisting microorganisms. Phylogenetic analyses confirmed HGT for 62 of these genes, including 34 derived from amoeba-resisting bacteria and 28 from giant viruses. Of the transferred genes, 48 were classified as potentially having virulence-associated traits in AK, including mannose-binding proteins (for adhesion), serine and metalloproteases (host cell degradation), phospholipases (host cellular degradation), as well as HSPs and antioxidant enzymes [52].

The presence of viral major capsid protein (MCP) genes through probable HGT between the host *A. castellanii* strain Neff and giant viruses, such as *Mollivirus,* was reported in three studies [53,62,68]. An experimental co-culture study identified approximately 273 proteins in the *A. castellanii* Neff strain that may have been acquired via HGT from intracellular giant viruses [53]. The host *A. castellani* was hypothesized to have acquired histone genes (H1, H2A, H2B, H3, and H4), DNA polymerase δ (B-family), Ran GTPase, and major capsid proteins via HGT from Medusavirus [62]. Horizontal acquisition of the tRNA-guanine transglycosylase (TGTase) gene from *Chlamydiae* was identified in *Acanthamoeba castellanii* [55].

Two studies reported the transfer of genes between intracellular microorganisms within the *Acanthamoeba* host [55,56]. The intracellular bacterium *Candidatus Babela massiliensis* appeared to have acquired a TGTase gene from *Chlamydiae* during its intracellular survival within the *Acanthamoeba* host [55]. Approximately 1338 genes of chlamydial origin were identified as having been transferred to *Megavirus chiliensis* during co-residence within an *Acanthamoeba* species host [56].

### 3.5. Effect of Intracellular Microbes on the Pathology of Acanthamoeba

Five studies (26.3%) analysed the presence of intracellular microorganisms on the pathological effects of the host *Acanthamoeba.* In an experimental cytopathogenicity study, *Acanthamoeba* species recovered from clinical and environmental sources harbouring unculturable intracellular bacteria had enhanced cytopathogenic effects on human embryonic tonsillar fibroblasts compared with uninfected *Acanthamoeba* counterparts [59]. In another experimental study using EpiCorneal cells (Cor-100-AFAB-; MatTek, Ashland, MA, USA), which is a three-dimensional human corneal tissue model, a novel endosymbiont closely related to *Mycobacterium* species was identified in *A. polyphaga* ATCC 50495 and *A. castellanii* 50493. Infection with *Acanthamoeba* isolates harbouring *Mycobacterium*-related species caused increased corneal epithelial damage and elevated pro-inflammatory cytokines (TNF-α, IL-1β, IL-6). This was speculated to occur along with upregulation of the epithelial injury/oxidative stress marker CuZn-SOD, providing evidence that intracellular microbes can enhance *Acanthamoeba* pathology [69].

Among eight clinical *Acanthamoeba* genotype T4 isolates, 50% harboured bacterial endosymbionts, including *Stenotrophomonas maltophilia* and *Achromobacter* spp., while 25% contained fungal endosymbionts such as *Gloeotinia* spp. Pathogenicity assays showed that 62.5% of endosymbiont-containing isolates exhibited increased virulence as compared to the isolates without endosymbionts, evidenced by osmo-tolerance, thermo-tolerance, and cytopathic effects on Vero cells [30]. Similarly, a study from Iran reported that among 15 clinical *Acanthamoeba* genotype T4 isolates, 60% harboured intracellular microorganisms, including bacteria, fungi, and human adenovirus. Isolates containing endosymbionts exhibited significantly higher osmo- and thermo-tolerance, increased trophozoite proliferation, and pronounced cytopathic effects on Vero cell monolayers, leading to complete cell destruction within 72 h [70] (Table 3).

On the other hand, predation by *Acanthamoeba* can increase the virulence for *C. neoformans*. During predation, *C. neoformans* undergoes polysaccharide capsule enlargement, increased melanin production, elevated extracellular urease secretion, and increased cell size [71]. The virulence of *Cryptococcus* is linked with its resistance to phagocytosis, but if engulfed is also able to survive and proliferate within the mature phagolysosome [72]. *Cryptococcus* virulence has been speculated to be linked to its ability to behave as a facultative intracellular pathogen, enabling it to evade the host immune system and produce virulence factors [73].

### 3.6. Antimicrobial Resistance Gene Transfer Within and Between Endosymbionts and Acanthamoeba

The capability of *Acanthamoeba* to harbour multiple intracellular microorganisms suggests that interactions among these microbes may occur, resulting in highly complex and heterogeneous effects on *Acanthamoeba* pathogenesis [8]. Three studies (15.7%) analysed HGT involving antimicrobial resistance genes between *Acanthamoeba* species and their intracellular microbes. The increased spread of antimicrobial resistance genes and virulence factor genes, along with the emergence of pathogenic antibiotic-resistant bacteria, is largely attributed to HGT [74]. In phylogenetic and rhizome gene mosaic analyses of ocular *Acanthamoeba* strains, genomic exchanges between *Acanthamoeba* and its intracellular microbes were shown including AMR genes such as *adeF*, *amrA*, and *amrB* [63]. These exchanges may contribute to drug resistance in *Acanthamoeba*.

In an experimental setup, the RP4 plasmid (carrying antibiotic resistance determinants) was transferred from a donor *P. putida* strain into the soil microbiome. Whilst subsequent exposure of the soil to *A. castellani* reduced the overall plasmid abundance, it increased the conjugation frequency and upregulated conjugation-associated genes in the microbiome, highlighting protozoa as ecological drivers of ARG dissemination [60]. Genetic exchange of AMR genes between intracellular bacteria within *Acanthamoeba* hosts also has been reported. Accordingly, the transfer of the *blaVIM-2* gene between *P. oleovorans* and *P. aeruginosa* was approximately 12 times higher in the presence of *Acanthamoeba* than in its absence [66] (Table 4).

### 3.7. Number of Genes Horizontally Transferred to the Host Acanthamoeba

**Figure 5:**
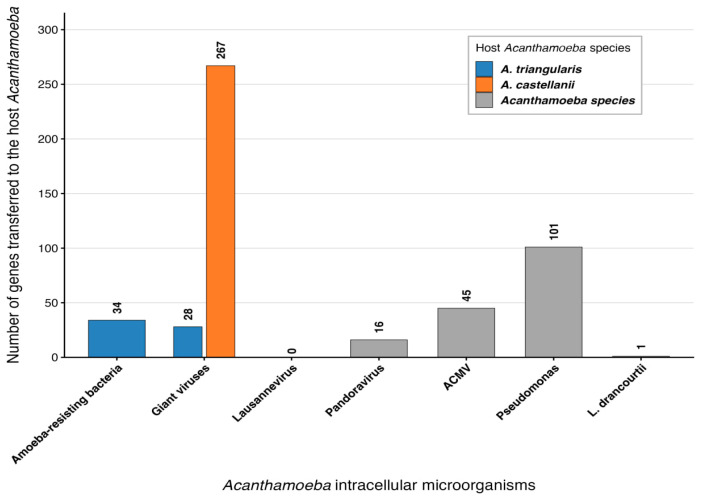
Bar graph showing the number of genes horizontally transferred to the host *Acanthamoeba* species from different intracellular microorganisms.

Five studies reported the number of genes that were potentially horizontally transferred from intracellular microorganisms to the *Acanthamoeba* host [52,53,61,63,67]. The highest number of HGT events was reported for intracellular giant viruses, with approximately 267 gene transfer protein markers identified in the *A. castellanii* Neff strain [53]. The second-highest number of gene transfer events was reported for *Pseudomonas* sp. in *Acanthamoeba* species, with an estimated 101 genes acquired through horizontal gene transfer [52]. However, in a long-term co-culture experiment between *Lausannevirus* and *A. castellanii* ATCC 30010, no genes were transferred to the host *Acanthamoeba* [61]. In a whole-genome sequencing study, only one malate synthase gene appeared to have been transferred from intracellular *Legionella. drancourtii* to *A. castellanii* [67] (Figure 5).

## 4. Discussion

This review systematically analysed 19 published studies on HGT between *Acanthamoeba* and its intracellular microorganisms, as well as the impact of these endosymbionts on *Acanthamoeba* pathogenicity and virulence. Whole-genome comparative genomics, followed by phylogenetic tree analysis, represents the most commonly used laboratory methods for identifying HGT events between *Acanthamoeba* and its intracellular microorganisms [52,64,67].

Potential pathogenic amoeba-resisting intracellular microorganisms have been reported to transfer genes to the host *Acanthamoeba* through HGT [52,53]. The phagotrophic feeding mechanism of *Acanthamoeba* promotes continual exposure to foreign DNA, and some microbes have been able to circumvent killing within the amoeba. These together can create favourable conditions for HGT [51]. Free-living amoebae can therefore act as genetic melting pots, shaping microbial evolution, and play a role as a training ground for adaptation to life in eukaryotic cells, persistence, and pathogenicity of microorganisms [75]. In addition, the evolutionary adaptation of intracellular microorganisms within *Acanthamoeba* may facilitate their expansion into mammalian hosts, thereby contributing to the emergence of various infectious diseases and increasing bacterial resistance to destruction by macrophages [76,77].

Bacteria possess mechanisms that allow them to evade killing by *Acanthamoeba*. For example, the Dot/Icm type IV secretion system genes, found in *Legionella pneumophila*, encodes bacterial effector proteins, which interfere with lysosomal fusion, phagosome maturation, and vesicle acidification, thereby protecting the bacteria from intracellular destruction [28]. Moreover, in addition to serving as environmental reservoirs for known human intracellular pathogens, *Acanthamoeba* may also act as a source of emerging bacterial pathogens [77]. As *Acanthamoeba* is widespread in environmental water and soil, HGT occurring within *Acanthamoeba* has public health significance, as it can select for microorganisms that survive within eukaryotic cells, which may, in turn, increase their potential to infect mammalian hosts [75,76]. However, not all intracellular microorganisms living within *Acanthamoeba* appear to undergo HGT, indicating that this process likely depends on specific biological and evolutionary conditions [61].

Bidirectional transfer of virulence genes such as metalloproteases, cysteine proteases, laminin-binding proteins, and heat shock proteins between amoeba-resisting microorganisms, including giant viruses and the *Acanthamoeba* species, has been reported [52]. Such interactions have been reported to have important clinical significance in pathogenesis, as the exchange of genes may not only alter genome content, but may also enhance corneal infection through increased host–cell damage and altered susceptibility of *Acanthamoeba* to anti-amoebic drugs [78]. *Acanthamoeba* corneal infection and pathology are mainly related to its ability to adhere to epithelial cells, which is enhanced by adhesins, including mannose-binding proteins and laminin-binding proteins [1]. Heat shock proteins are important for enabling organisms to survive and adapt to higher temperatures and maintain their metabolic activity within the host [79]. Therefore, genomic analysis is a valuable strategy for identifying and characterizing potential targets for the development of new therapeutic approaches [79,80]. In addition, the association between pathogenicity and increased extracellular protease shows that pathogenic *Acanthamoeba* utilizes protease to increase invasion and damage to the host cell [81]. This is supported by the observation that proteases produced by *Acanthamoeba* produce an increase in cytopathic effects compared to those that are not pathogenic, killing host cells and degrading the epithelial basement membrane as well as the stromal matrix, and facilitating progression into the deeper layers of the cornea [82]. This suggests that the presence of intracellular microorganisms in *Acanthamoeba* can further increase its pathogenicity. Detailed molecular and cellular studies are required to clarify the mechanisms through which amoeba-resisting microorganisms affect the virulence of *Acanthamoeba* and their interaction. The transfer of genes between intracellular microorganisms within the *Acanthamoeba* host have been reported [55,56]. The close intracellular survival of different microbes within the same *Acanthamoeba* cytoplasmic environment creates favourable conditions for HGT within the endosymbionts [83]. An additional mechanism that may contribute to HGT between *Acanthamoeba* and its intracellular microbes is extracellular vesicle (EV)-mediated genetic exchange. Extracellular vesicles are membrane-bound particles released by cells that can transport diverse molecular cargo, including DNA, RNA, proteins, and lipids [35]. Increasing evidence suggests that intracellular pathogens can modify the composition of host-derived EVs and exploit them for intercellular communication and the dissemination of virulence-associated molecules [84]. EV-mediated transfer of genetic material has been documented in several host–pathogen and parasite systems, indicating that these vesicles may facilitate the movement of nucleic acids between cells [85]. Although no studies have directly demonstrated EV-mediated horizontal gene transfer between *Acanthamoeba* and its intracellular microbes, infected amoebae may potentially release EVs containing microbial genetic material that could be taken up by neighbouring amoebae [35,85].

Additionally, gene exchange within the amoebal cytoplasmic environment may contribute to the development of highly sophisticated virulence strategies, enhance intracellular survival, and possibly promote antimicrobial resistance [86]. Therefore, *Acanthamoeba* can serve as both a host and a vehicle for microorganisms, with important implications for public health, as exposure to *Acanthamoeba* may also increase the virulence of some pathogens [75].

The effect of intracellular microorganisms on the pathological effects of *Acanthamoeba* was reported in this review. Through increased secretion of cell lysis factors, altered gene expression, and enhanced inflammatory responses, intracellular microorganisms can modulate host *Acanthamoeba* pathogenicity [76,87]. The intracellular replication of bacteria has been shown to enhance the virulence of *Acanthamoeba*, whereas the *Acanthamoeba* protects these bacteria against chlorine and other biocides [87].

An increase in the drug resistance of *Acanthamoeba* was observed as a result of the genomic exchange of genes, including *adeF*, *amrA*, and *amrB*, between the *Acanthamoeba* host and its intracellular microbes [63]. *amrA* and *amrB* are well-characterized genes that confer resistance to aminoglycoside and macrolide antibiotics and belong to the AMR gene family of resistance nodulation division (RND) antibiotic efflux pumps, which can decrease intracellular antibiotic concentration and make *Acanthamoeba* less susceptible to neomycine azithromycin [88]. Amoebae serve as a melting point for the genetic exchange of antimicrobial resistance markers (ARMs), and HGT within these hosts is considered a significant driver of antimicrobial resistance [89].

## 5. Conclusions and Recommendations

The findings of this systematic review indicate that *Acanthamoeba* serves as an important habitat for HGT among intracellular microorganisms. HGT has been identified between *Acanthamoeba* and other intracellular microorganisms, including bacteria and giant viruses. Genetic transfer can occur among intracellular microorganisms coexisting within the *Acanthamoeba* host. Intracellular microorganisms in *Acanthamoeba* were associated with increased pathogenic potential of *Acanthamoeba*. The transfer of VAGs and ARGs indicates that intracellular microorganisms within *Acanthamoeba* contribute to increased host pathogenicity. Hence, *Acanthamoeba* plays an important role in the evolution of virulence and antimicrobial resistance among intracellular microorganisms.

More studies are needed to indicate the molecular mechanisms underlying HGT of antimicrobial resistance and VAGs between intracellular microorganisms and *Acanthamoeba*. Further surveillance of clinical and environmental samples is needed to map HGT of virulence and ARGs between the host *Acanthamoeba* and its intracellular microbes. Genomic, transcriptomic, and functional studies, including examining EV-mediated genetic transfer, are needed to assess the biological significance of horizontally transferred genes and their role in virulence and antimicrobial resistance. Improved emphasis on the role of *Acanthamoeba* in HGT of virulence and ARGs increases our understanding of treatment failure, pathogenesis, and the spread of antimicrobial resistance genes.

## 6. Strengths and Limitations of the Systematic Review

One of the main strengths of this systematic review is that it synthesized the available evidence on HGT between *Acanthamoeba* and its intracellular microorganisms across a broad range of microbial groups, including bacteria, fungi, and viruses. Moreover, the included studies have a good quality, which increases the reliability of the findings of this systematic review. In addition, this systematic review reports on the impact of the intracellular survival of microbes in *Acanthamoeba* on its pathogenicity and virulence. The major limitation of this systematic review was the inability to perform a meta-analysis because of substantial heterogeneity in the variables reported across the included studies. Moreover, most of the studies use WGS for detecting HGT between *Acanthamoeba* and its intracellular microorganisms and this may have introduced bias.

## Figures and Tables

**Table 2:** Genes transferred between the *Acanthamoeba* intracellular microorganism and the host *Acanthamoeba* species and effect on the virulence of microorganisms.

Study Author/Year	Source of Sample	Host *Acanthamoeba* Species	*Acanthamoeba* Intracellular Microbes	Samples with Intracellular Microbes	Genes Transferred	Effect on Virulence	HGT Detection Method
Gu et al., 2022 [22]	Clinical and environmental	*Acanthamoeba* species genotype T3 and T4, including *A. castellanii*, *A. polyphaga*, *A. triangularis*	*Pseudomonas* spp., *Mycobacterium* genus, *Rickettsia* isolates, *L. pneumophila*, *Chlamydia trachomatis*, *Cryptococcus depauperatus*, *C. neoformans*, *Pandoraviruses*, *A. castellanii* medusavirus	7 of 7	VAGs (metalloproteases, cysteine proteases, LBP, HSP), metabolic and signaling genes, viral homologs	HGT with ARMs, especially *Pseudomonas* species, and enrichment of virulence genes (LBP, proteases, HSPs)	Whole-genome comparative genomics
Hasni et al., 2020 [52]	Clinical (corneal)	*Acanthamoeba triangularis* (genotype T4)	*Chlamydiae*, *L. pneumophila*, *Acinetobacter*, *Pseudomonas* species, and giant viruses (*Pandoraviridae*, *Medusavirus*, *Mimiviridae*, *Marseillevirus*, *Pithovirus*, *Mollivirus*)	1 of 1	99 *A. triangularis* genes with best BLASTp hits to ARMs, phylogenetic analysis confirmed HGT for 62 genes (34 with amoeba-resisting bacteria, 28 with giant viruses)	Genomic analysis identified 48 VAGs associated with AK (including MBP, multiple serine and metalloproteases, phospholipases, HSP, antioxidant enzymes)	Whole-genome comparative genomics
Maumus & Blanc, 2016 [53]	Laboratory co-culture experment	*A. castellanii* strain Neff	Giant viruses (*Pandoravirus*, *Mimivirus*, *Marseillevirus*, *Pithovirus*, *Mollivirus*)	1 of 1	267 LGT markers; viral genes including MCPs, ATPase, ligase	Not reported	BLASTP, phylogenetics, CDI, transcriptomics
Manna & Harman, 2016 [55]	Genome sequences from public databases	*A. castellanii*	*Chlamydiae* and *Candidatus babela massiliensis*	Not reported	*A. castellanii* acquired a TGTase gene from Chlamydiae. Candidatus Babela massiliensis acquired its TGTase from *Chlamydiae* inside *Acanthamoeba.*	Affect tRNA modification pathways, influence amoebal metabolism, and are considered a potential drug target because of similarity to virulence-associated TGTases	BLASTp, phylogenetic reconstruction, sequence similarity analysis
Mueller et al., 2017 [61]	*A. castellanii* ATCC 30010 culture collection	*A. castellanii* ATCC 30010	*Lausannevirus*, *Estrella lausannensis*	1 of 1	No evidence for gene transfer having occurred	Not reported	LGT was investigated by BLASTN
Takemura 2020 [62]	Environmental (hot spring water)	*A. castellanii*	*Medusavirus*	Not reported	Histone genes (H1, H2A, H2B, H3, H4), DNA polymerase δ (B-family), Ran GTPase, and MCP	Not reported	Comparative genomics & molecular phylogenetics
Ling et al., 2024 [63]	Clinical (ocular AK isolates) and Environmental (water, soil)	*Acanthamoeba* species	Bacterial endosymbionts: *Burkholderia*, *Klebsiella*, *Pseudomonas*, *Chlamydia*, *Mycobacterium*, *L. pneumophila*, *V. cholerae*, *Aspergillus* species, *Pandoravirus*, and *A. castellanii medusavirus*	48 strains total, 19 clinical ocular strains	adeF, amrA, amrB (ARGs transferred from *Burkholderia* to *Acanthamoeba*)	Indirectly suggested this may have enhanced drug resistance linked to treatment failure in AK	Comparative genomics, BLASTp (CARD), phylogenetic trees, bootstrap-validated HGT, rhizome or mosaic gene analysis
Erber et al., 2021 [64]	Environmental	*A. castellanii*	*Desulfovibrio* species and related Proteobacteria	Not applicable	ntr4 (A-adding tRNA nucleotidyltransferase)	Not reported	Sequence similarity analysis, phylogenetic network analysis, and recombinant protein functional validation
Rolland S., 2020 [65]	*Acanthamoeba castellanii* ATCC 30010 culture collection	*A. castellanii* ATCC 30010	*prokaryotes and belonging to the phyla of Chlorobacteria,* cyanobacteria, *and Firmicutes*	1 of 1	lateral transfer of the ACA1_384820 gene (encodes a putative GNAT-family N-acetyltransferase) from prokaryotes	Not reported	BLASTp against NCBI nr showing best hits in bacteria
Sarink et al., 2025 [66]	*Acanthamoeba castellanii* ATCC strain culture collection	*A. castellanii* ATCC 30010	*P. oleovorans* (plasmid donor) and *P. aeruginosa* strain 957 (recipient); an additional 18 *P. aeruginosa strains tested*	7 co-culture experiment	blaVIM-2 (plasmid-encoded carbapenem-resistance gene)	Not reported	Confocal microscopy, MALDI-TOF
Watanabe et al., 2018 [56]	Environmental	*Acanthamoeba* species	*Chlamydiae* and *Mimiviridae*, *Megavirus chiliensis*	Not reported	1338 genes of the *Chlamydiae* were found to be shared with the *Megavirus chiliensis*	Not reported	Genomic annotation with BLAST analysis using RAST, functional annotation was also performed using the KEGG and phylogenetic analysis
Lin et al., 2025 [60]	Environmental (agricultural soil)	*A. castellanii*	*P. putida* mixing experiment Predation	Not applicable	Plasmid-borne genes on RP4 plasmid (blaTEM (β-lactam), tetA (tetracycline), aph(3′)-Ib (kanamycin), gfp reporter	Virulence genes related to ARGs were detected: protozoa selected transconjugants carrying virulence factor genes (VFs) (tlyC, cya, acrB adjacent to intI1)	Fluorescence-activated cell sorting (FACS), qPCR, RT-qPCR, metagenomics
Moliner et al., 2009 [67]	Environmental water	*A. castellanii*	*L. drancourtii*	1 of 1	Malate synthase gene (From *L. drancourtii* to Acanthamoeba)	Not reported	Whole-genome sequencing, BLASTp/tBLASTn, reciprocal BLAST, phylogenetic analysis
Matthey-Doret et al., 2022 [68]	Experimental laboratory co-culture	*A. castellanii* strains Neff and C3	Experimental infection of *A.castellani* with *L. pneumophila*	1of 1	*A. castellani* strain Neff carries the MCP gene with strong similarity to *Mollivirus*	Not assessed	Comparative genomics

Key: Acanthamoeba keratitis = AK, antimicrobial resistance genes = ARGs, amoeba-resisting microorganisms = ARMs, Verona integron-encoded metallo-β-lactamase = blaVIM-2, Comprehensive Antibiotic Resistance Database = CARD, horizontal gene transfer = HGT, heat-shock proteins = HSPs, Kyoto Encyclopedia of Genes and Genomes = KEGG, laminin-binding proteins = LBP, lateral gene transfer = LGT, mannose-binding proteins = MBP, major capsid proteins = MCPs, Rapid Annotation using Subsystem Technology = RAST, tRNA-guanine transglycosylase = TGTase, virulence-associated genes = VAGs.

**Table 3:** The effect of intracellular microorganisms on the pathology of *Acanthamoeba*.

Study Author/Year	Host *Acanthamoeba* Species	*Acanthamoeba* Endosymbionts	Effect on the Pathology of *Acanthamoeba*
Fritsche et al., 1998 [59]	*Acanthamoeba* species	*Chlamydia*-like Gram-negative coccus, Gram-negative rods	Endosymbiont-infected *Acanthamoeba* showed increased cytopathic effect on human embryonic tonsilar fibroblast
Fu et al., 2021 [71]	*A.* *castellanii*	*Cryptococcus neoformans*	*C. neoformans* expresses virulence, mutations in the gene encoding the oligopeptide transporter (CNAG_03013; OPT1)
Purssell et al., 2017 [69]	*A. castellanii*, *A. polyphaga*, *A. culbertsoni* (ATCC)	Holosporaceae (Rickettsiales) in *A. polyphaga* 30173, *Mycobacterium* species in *A. polyphaga* 50495, *C. procabacter* species OEW1 and *Parachlamydia* species OEW1 in *Acanthamoeba* PRA-220	Infection of EpiCorneal tissue with *A. castellanii* 50493 and *A. polyphaga* 50372 increased TNF-α, IL-1, IL-6 and CuZn-SOD and caused cytopathic changes
Soleymani et al., 2024 [30]	*Acanthamoeba* species genotype T4	*S. maltophilia*, *Achromobacter* species, uncultured fungus, *Gloeotinia* species	5/8 isolates were highly pathogenic (thermo-/osmo-tolerant and CPE)
Hajialilo et al., 2019 [70]	*Acanthamoeba* T4 genotype	*E. coli*, *Achromobacter* species, *P. aeruginosa*, *S. maltophilia*, *Microbacterium* species, *Brevibacillus* species, *Brevundimonas* species, *Aspergillus* species, human adenovirus (HADV)	Isolates with endosymbionts (ICS2 *E. coli*; ICS7 with bacterial, fungal, and viral endosymbionts) showed higher pathogenicity and more severe CPE on Vero cells than the endosymbiont-free isolate ICS9

**Table 4:** Studies reporting transfer of antimicrobial resistance genes between and within endosymbionts and *Acanthamoeba*.

Study Author/Year	Host *Acanthamoeba* Species	*Acanthamoeba* Endosymbionts	AMR Genes Detected
Ling et al., 2024 [63]	*Acanthamoeba* species (T4 genotype)	Bacterial endosymbionts: *Burkholderia*, *Klebsiella*, *Pseudomonas*, *Chlamydia*, Mycobacterium, *L. pneumophila*, *V. cholerae*, Aspergillus spp.; giant viruses: *Pandoravirus*, *A. castellanii medusavirus*	Unidirectional HGT from *Burkholderia* to *Acanthamoeba* involving RND efflux pump genes (adeF, amrA, amrB)
Lin et al., 2025 [60]	*A. castellanii*	*P. putida* mixing experiment predation	β-lactam(blaTEM), tetracycline(tetA), aminoglycoside [APH(3′)-Ib], ARG classes detected in transconjugants via metagenomics
Sarink et al., 2025 [66]	*A. castellanii* ATCC 30010	*P. oleovorans* (plasmid donor) and *P. aeruginosa* strain 957 (recipient), additional 18 *P. aeruginosa* strains tested	blaVIM-2

Key: ARG = antimicrobial resistance gene, ATCC = American type culture collection, bla VIM-2 = Verona integron-encoded metallo-β-lactamase, HGT = horizontal gene transfer, RND = resistance-nodulation-cell division.

## Data Availability

No new data were created or analysed in this systematic review.

## Supplementary Materials

The following supporting information can be downloaded at: https://www.mdpi.com/article/10.3390/pathogens15060610/s1, Table S1: The included studies’ quality appraisal in the systematic review of The Implication of Horizontal Gene Transfer between Acanthamoeba and its intracellular microbes on Pathogenicity: 2026; Table S2: Data extraction sheet of included studies. Supplementary File S1 PRISMA 2020 Checklist.

## Abbreviations

The following abbreviations are used in this manuscript:

AK	Acanthamoeba keratitis
ARMs	Amoeba-resisting microorganisms
ARVs	Amoeba-resisting viruses
GAE	Granulomatous amoebic encephalitis
HGT	Horizontal gene transfer
HSP	Heat-shock proteins
KEGG	Kyoto Encyclopedia of Genes and Genomes
LBP	Laminin-binding proteins
MBP	Mannose-binding proteins
MCP	Major capsid protein
PRISMA	Preferred Reporting Items for Systematic Reviews and Meta-analyses
RAST	Rapid Annotation using Subsystem Technology
TGTase	tRNA-guanine transglycosylase
VAGs	Virulence-associated genes
