# Supplementary material for: The Implication of Horizontal Gene Transfer Between Acanthamoeba and Its Intracellular Microbes on Pathogenicity: A Systematic Review
Source: Pathogens. 2026 Jun 8;15(6):610. doi: 10.3390/pathogens15060610 (PMC13305018; doi:10.3390/pathogens15060610)
Supplement: Supplementary file 1 [file pathogens-15-00610-s001.zip › Table S1The included studies' quality appraisal in the systematic review.pdf]

Table S1: The included studies' quality appraisal in the systematic review of The Implication of Horizontal Gene Transfer between *Acanthamoeba* and its intracellular microbes on Pathogenicity: 2026

Number	Author/year	Source of samples for Acanthamoeba	Study quality
1	Gu et al., 2022	Corneal scraping specimen	Good
2	Hasni et al., 2020	ATCC strain	Good
3	Maumus & Blanc, 2016	Laboratory co-culture experiment	Good
4	Manna & Harman, 2016	Genomic sequences from the public database	Good
5	Mueller et al., 2017	ATCC strain	Good
6	Takemura 2020	Environmental samples from hot spring water	Good
7	Ling et al., 2024	Clinical (ocular AK isolates) and Environmental (water, soil)	Good
8	Erber et al., 2020	Environmental (genome-based laboratory strain analysis)	Good
9	Rolland S.,2020	ATCC strain	Good
10	Sarink et al., 2025	ATCC strain	Good
11	Watanabe et al., 2018	Environmental samples	Good
12	Lin et al., 2025	Environmental sample	Good
13	Moliner et al., 2009	Environmental sample	Good
14	Matthey-Doret et al., 2022	Environmental	Good
15	Fritsche et al., 1998	Clinical corneal and environmental samples	Good

16	Fu et al., 2021	Clinical and environmental strains of Acanthamoeba	Good
17	Purssell et al., 2017	ATCC strains	Good
18	Soleymani et al., 2024	Clinical isolates	Good
19	Hajialilo et al., 2019	Clinical corneal scrapes and contact lenses from keratitis patients	Good
